# Cognitive and Mood Effects of a Nutrient Enriched Breakfast Bar in Healthy Adults: A Randomised, Double-Blind, Placebo-Controlled, Parallel Groups Study

**DOI:** 10.3390/nu9121332

**Published:** 2017-12-07

**Authors:** David O. Kennedy, Emma L. Wightman, Joanne Forster, Julie Khan, Crystal F. Haskell-Ramsay, Philippa A. Jackson

**Affiliations:** Brain, Performance and Nutrition Research Centre, Northumbria University, Newcastle-upon-Tyne NE1 8ST, UK; emma.l.wightman@northumbria.ac.uk (E.L.W.); joanne.forster@northumbria.ac.uk (J.F.); julie.khan@northumbria.ac.uk (J.K.); crystal.haskell-ramsay@northumbria.ac.uk (C.F.H.-R.); philippa.jackson@northumbria.ac.uk (P.A.J.)

**Keywords:** vitamin, l-theanine, l-tyrosine, caffeine, cognitive, mood

## Abstract

Objectives: Few previous studies have assessed the effects of concomitant administration of multiple potentially psychoactive nutrients. Methods: 95 healthy adult participants consumed either a nutrient enriched breakfast bar (containing α-Linolenic acid, l-tyrosine, l-theanine, vitamins, minerals and 21.5 mg of caffeine) or an isocaloric, macronutrient matched control bar for 56 days. Cognitive function and mood were assessed pre-dose and at 40- and 160-min post-dose on the 1st and 56th day of the intervention period. Results: The results demonstrated acute effects of treatment across post-dose assessments on both assessment days in terms of alertness, and on tasks assessing attention, working and episodic memory and executive function, including cognitively demanding Serial subtraction and Rapid Visual Information Processing tasks. There were no evident chronic effects independent of the breakfast bars’ acute effects. Discussion: These results demonstrate that a nutrient enriched breakfast bar with low caffeine content can exert striking beneficial effects on acute cognitive function and alertness.

## 1. Introduction

Optimal brain function requires adequate dietary intakes of a wide range of essential and non-essential nutrients, including vitamins, minerals, amino-acids and polyunsaturated fatty acids. However, cross-sectional data suggests that a sizeable minority of the populations of developed countries exhibit biochemical deficiencies in one or more of these nutrients that would predispose them to specific or general diseases related to a lack of that nutrient. For instance, in terms of vitamins/minerals alone, governmental evidence from the UK [1,2] suggests that deficiency/depletion levels for non-elderly adults are approximately 4% for vitamin C, 5% for vitamin B_12_ and approximately 66% for vitamin B_2_. Some 15.5% of females are also deficient in iron (plasma ferritin). It is notable that only a small minority of adults meet government minimum guidelines for fruit and vegetable and oily fish consumption [2]. 

These figures are hardly surprising given that governmental intake recommendations (e.g., Recommended Dietary Allowance (RDA) and Recommended Dietary Intake (RDI)), which many fail to achieve from contemporary diets, are generally set at the minimum level required to avoid disease states in most of the population. Naturally, the optimal level of any nutrient will not merely be the level that prevents an increased likelihood of disease, suggesting that a considerable proportion of the populations of developed countries have less than optimal nutritional status. Nutritional status is important because epidemiological evidence suggests that greater consumption or higher circulating levels of a wide range of nutrients are related to improved cognitive function, or an attenuation of age-related cognitive decline or risk of dementia. For instance, this relationship has been highlighted for vitamins B_6_, B_9_ and B_12_ [3,4], vitamin C [5,6], vitamin A [6], vitamin E [7], iron [8], caffeine [9], foods rich in omega-3 polyunsaturated fatty acids (omega-3 PUFAs) [10,11], and polyphenols [12,13,14]. A similar relationship is also evident with respect to the general nutrient adequacy of an individual’s diet [9].

Given the above it is not surprising that, conversely, supplementation with several of the above nutrients over a period of time may benefit psychological functioning. For instance, accumulating evidence in normal, healthy adults suggests that supplementation with multi-vitamins/minerals can improve cognitive function, enhancing performance during multi-tasking and on difficult mental arithmetic tasks, whilst improving aspects of psychological state (e.g., subjective stress, mood, and fatigue) [15,16,17]. In contrast, the improvements seen in cognitive function (attention, memory and learning) following iron supplementation appear to depend on initial iron status where those with initially low levels show the greatest increases [18]. Polyphenols, such as from cocoa [19,20,21], are reported to improve performance on cognitive tasks and, extended supplementation of omega-3 is observed to improve mood, verbal fluency and memory and reaction time [22,23].

A number of key nutrients have also been shown to exert beneficial effects on brain function following a single dose. The obvious example here is caffeine, which reliably increases alertness/arousal and the performance of attention tasks [24]. However, other psychoactive nutrients include the tea specific amino-acid l-theanine; which can improve vigilance (on a rapid processing task), reaction time and working memory when consumed with caffeine [25] and essential amino-acids such as l-tyrosine; which improve complex cognitive processes like word association, cognitive flexibility and updating [26,27,28,29]. Intriguingly, evidence also suggests that single doses of vitamins have bioactive properties. For instance a single dose of vitamin B_9_, vitamin C, or vitamin E can increase peripheral vasodilation as measured by flow mediated dilation [30,31,32,33,34,35] and single doses of calcium [36], calcium combined with vitamin D [37] and a multi-vitamin/mineral [38] can increase fat oxidation. Of more relevance to the brain, improvements in attention and semantic memory task performance have been demonstrated following the first dose of a multivitamin/mineral administered to children [39] and single doses of multi-vitamin/minerals have been shown to modify regional brain activity [40], cerebro-electrical activity [41] and cerebral blood-flow [38] using a variety of imaging techniques.

Taken together, these data suggest that many people may be deficient in multiple micronutrients and other beneficial nutritional compounds, and that this might contribute to aspects of poor health. Research suggests that cultures which are less likely to access sufficient levels of these compounds from dietary sources are more likely to supplement their diets to compensate for nutrient shortfalls [42]: In the US over half of adults consume supplements (predominantly multivitamins, calcium and fish oils) [43] compared to less than 10% in Mediterranean-diet consuming countries like Spain [44]. As such, dietary supplementation with a nutrient enriched food designed to provide an adults approximate daily minimum requirement of a broad range of essential and non-essential nutrients (including vitamins and minerals, omega-3 PUFAs and amino acids, plus a low dose of caffeine) may benefit multiple aspects of cognitive function and mood. This approach may offer a viable means of consuming essential nutrients for those who do not access these from the diet. This randomised, double-blind, placebo-controlled, parallel groups study therefore investigated the effects of such an intervention on the first day of supplementation and following 8 weeks administration, in healthy young adults.

## 2. Materials and Methods

### 2.1. Design

The study comprised a randomised, double-blind, placebo-controlled, parallel group comparison of the effects of acute (Day 1) and chronic (Day 56) consumption of a nutrient enriched breakfast bar in comparison to a macronutrient-matched control bar. Eight weeks was arbitrarily chosen as the supplementation period in the absence of any evidence to indicate what an “effective” period of time would be. The study received ethical approval from the Northumbria University Faculty of Health and Life Sciences Ethics Committee (reference: RE-HLS-13-131121-528de56935e26; date approved on 6 January 2014) and was conducted according to the Declaration of Helsinki (1975). All participants gave their written informed consent prior to their inclusion in the study.

### 2.2. Participants

**Figure 1:**
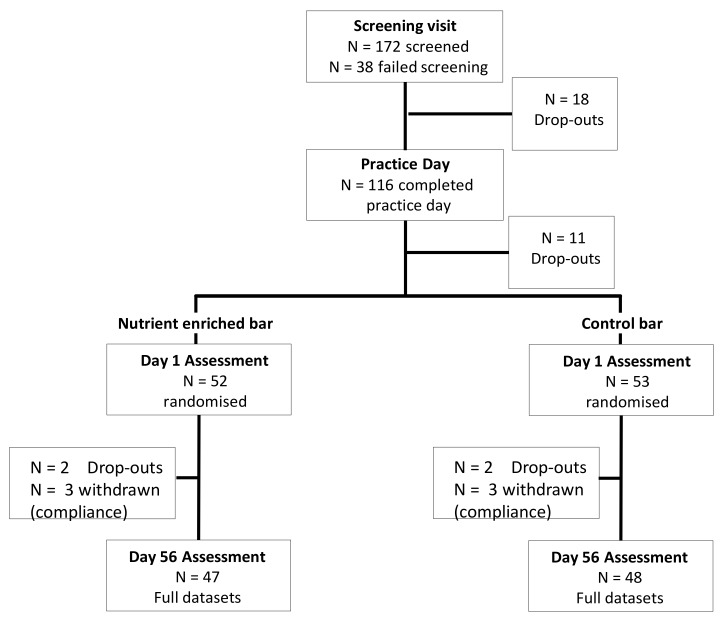
Flow-diagram showing participant dispositions throughout the study.

A total of 105 participants, aged 21 to 40 years, who self-reported themselves to be in good physical health were initially randomised. Following drop-outs (due to no further interest in the study) and withdrawals (due to lack of compliance with treatment consumption or adherence to task completion) the data from a total of 95 participants (48/47 placebo/active) were subsequently entered into the analysis. See Figure 1 for details of participant dispositions throughout the study. Given the multiple active ingredients, it was not possible to calculate a sample size which would encompass all components but approximately *N* = 50 per group was considered a reasonable number based on existing research trials in this area.

The exclusion criteria for the study included: inability to adequately perform the cognitive tasks, presence or history of any chronic disease, including neurological, developmental and mood disorders and self-reported sleep disturbance; use of dietary supplements, use of prescribed medications (with the exception of oral contraceptives, some topical skin treatments and occasional inhaler use); non-use of caffeine; food allergies; history of alcohol or drug abuse; or smoking. Participants with high blood pressure (>139/89 mm Hg) or a BMI > 35 and those that were pregnant/lactating were also excluded.

Demographics for the participants entered into the analysis are presented in Table 1.

### 2.3. Treatments

Treatments comprised one of two 50 g biscuit bars which were matched in terms of calories (220 Kcal), dietary fibre (4.7 g), sugars (8.6 g) and protein (9.6 g). 

The control breakfast-bar contained white flour, vegetable oil, sugar, almond flavouring and eggs. The nutrient enriched bar (Cognutria Foods Inc., Novato, CA, USA) contained whole wheat flour, peanuts, walnuts, almonds, whey protein, almond flour, ground flax meal, coconut flour, eggs, brown cane sugar, milk chocolate solids, dark cane sugar, cocoa powder, quinoa, ground coconut, coconut oil. In addition, the following, derived from natural extracts, were added: l-theanine, lysine, vitamins B_6_, B_9_, B_12_, zinc gluconate, magnesium citrate and caffeine. These ingredients delivered a total of 1.4 mg of vitamin A, 15 mg of vitamin E, 5 mg of vitamin B_5_, 1.9 mg of vitamin B_6_, 400 µg of vitamin B_9_, 500 µg of vitamin B_12_, 400 mg of magnesium, 1000 mg of calcium, 0.36 mg of iron, 11 mg of zinc, 1.4 g of omega-3 fatty acids (α-linolenic acid), 279.0 mg of l-tyrosine, 21.5 mg of caffeine, 59.2 mg of tryptophan, 39.3 mg of choline, 867 mg of lysine, 125 mg of l-theanine, and 7.3 mg of theobromine.

Control and nutrient enriched bars were delivered from the manufacturers identified only by a code and were boxed and distributed to participants according to the computer-generated randomisation schedule by a disinterested third party. To ensure that the researchers remained blinded as to which bar each participant consumed, the participants were asked to refrain from discussing any aspect of their biscuit with them and the researcher never viewed the biscuits; which were prepared for participants by a disinterested 3rd party. When required to make a forced choice at the end of the treatment period, just over half of all participants (60%) guessed the nature of their treatment correctly (with 49% accuracy in the nutrient enriched condition alone). These figures did not represent significantly higher than chance performance.

### 2.4. Cognitive and Mood Measures

**Figure 2:**
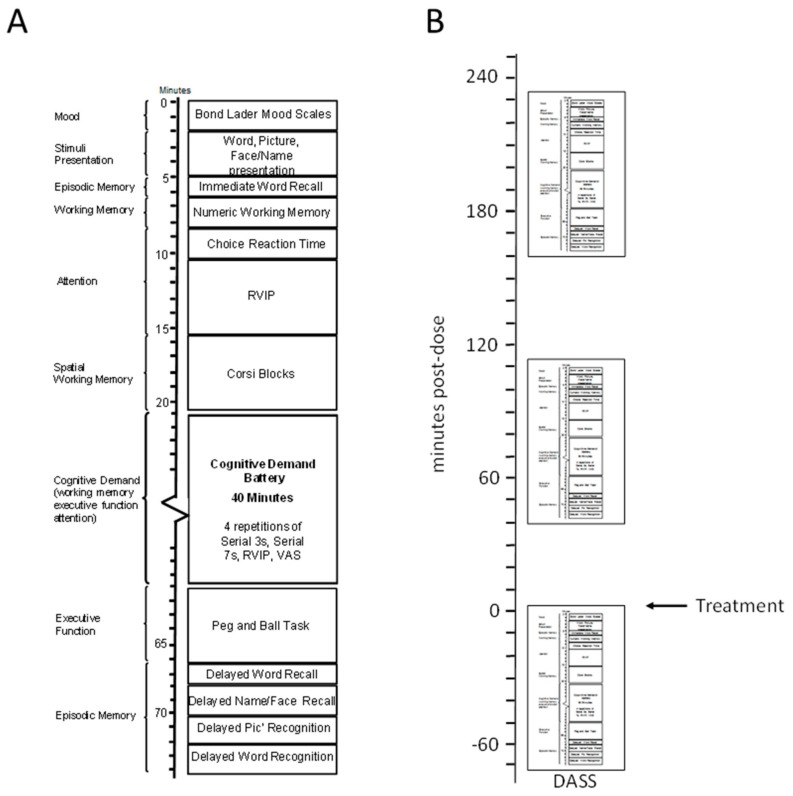
(**A**) The running order of the individual cognitive assessments. Tasks are shown in order of completion with approximate timings with on the left the “cognitive domain” assessed by the task; (**B**) Testing session timeline for the 1st (Day 1) and 56th (±2 days) day (Day 56) of treatment consumption. On each day participants arrived at ~8.30 a.m., completed the Depression Anxiety and Stress Scales (DASS) and one baseline cognitive assessment (as per panel A) and then consumed their treatment for that day. They then completed further assessments, commencing at 40 min post-dose and at 160 min post.

Performance was assessed using the Computerised Mental Performance Assessment System (COMPASS—Northumbria University, Newcastle upon Tyne, UK); a software platform for the presentation of classic and bespoke computerised cognitive tasks, with fully randomised parallel versions of each task delivered at each assessment for each individual. Tasks were presented on a laptop PC with responses made either via a four-button response box, mouse and cursor, the keyboard’s linear number pad or, for word recall only, pen and paper. The selection of standard, classic, cognitive tasks were specifically chosen to provide a broad assessment across all cognitive domains, including episodic memory, working memory, attention and executive function (Figure 2). Similar selections of tasks have previously been shown to be sensitive to a number of nutritional interventions [23,45,46,47]. Additionally, the Cognitive Demand Battery elements assessed working memory/executive function during a prolonged period of sustained cognitive processing. This approach, inculcating high cognitive demands for an extended period of time, has been shown to be sensitive to the effects of numerous nutritional interventions [19,48,49,50,51]. The tasks and other components of each assessment are described below in order of completion. The timelines of each assessment and the cognitive domains that individual tasks load upon are shown in Figure 2A.

#### 2.4.1. Picture Presentation

Fifteen colour photographic images of objects were presented sequentially on screen for the participant to remember at the rate of 1 every 3 s, with a stimulus duration of one second.

#### 2.4.2. Face Presentation

A set of twelve passport-style photographic images of people were presented sequentially in a random order to participants. A first and last name was assigned to each photograph and presented on the screen underneath the person’s face. Stimulus duration was three seconds, with a one-second inter-stimulus duration.

#### 2.4.3. Word Presentation

A unique set of fifteen words was presented. Words were selected at random from a large bank of words (MRC Psycholinguistic Database) matched for word length, frequency, familiarity and concreteness. Stimulus duration was one second, as was the inter-stimulus duration.

#### 2.4.4. Immediate Word Recall

The participant was allowed 60 s to write down as many of the words that were just presented as possible. The task was scored for number correct and errors.

#### 2.4.5. Numeric Working Memory

Five random digits from 1–9 were presented sequentially for the participant to hold in memory. This was followed by a series of 30 probe digits (15 targets and 15 distractors) for which the participant indicated whether or not it had been in the original series by a simple “yes” or “no” key press. The task consisted of 3 separate trials. Accuracy and mean reaction time for correct responses were recorded.

#### 2.4.6. Choice Reaction Time

An arrow appeared on the screen pointing to the left or to the right. Participants responded with a left or right key press corresponding to the direction of the arrow. There was a randomly varying inter-stimulus interval of between 1 and 3 s for a total of fifty stimuli. Accuracy and mean reaction time for correct responses were recorded.

#### 2.4.7. Rapid Visual Information Processing (RVIP) Task

During this 5 min task the participants monitored a continuous series of single digits (1–9) for targets of three consecutive odd or three consecutive even digits. The digits were presented on the computer screen at the rate of 100 per minute in a pseudo-random order, with the participant responding to the detection of a target string with a space bar key press. Eight target strings were presented in each minute. The task was scored for number of correctly identified target strings and average reaction time for correct detections.

#### 2.4.8. Corsi Blocks Task

In this task nine identical blue squares appeared on screen in non-overlapping random positions. A set number of blocks changed colour from blue to red in a randomly generated sequence. The cursor was locked in position until the entire sequence had been presented, at which point the participants were instructed to repeat the sequence by clicking on the blocks using the mouse and cursor. The task was repeated five times at each level of difficulty. The sequence span increased from 4 upwards, until the participant could no longer correctly recall the sequence, resulting in a span measure of nonverbal working memory, calculated by averaging the level of the last three correctly completed trials.

#### 2.4.9. Cognitive Demand Battery

In this instance, four repetitions of a 10 min computerised “Cognitive Demand Battery” (total running time ~40 min) were completed before the delayed components of the episodic memory tasks. The battery comprises four components: A Serial 3s subtraction task (2 min), Serial 7s subtraction task (2 min), Rapid Visual Information Processing (RVIP-5 min) and a “mental fatigue” visual analogue scale. For the serial subtraction tasks, participants subtracted either 3 or 7 consecutively from a randomly generated number between 800 and 999 for the duration of the task, entering their responses on the keyboard’s linear number pad. These tasks were scored for the number of correct subtractions and the number of errors. The RVIP task was as described above. Following each completion of the three tasks participants rated their current subjective “mental fatigue” by using the cursor to position a cross on a visual analogue scale anchored “not at all” and “extremely” at the ends. The Cognitive Demand Battery has previously been used effectively to investigate the effects of various nutritional interventions on cognitive performance and mental fatigue during periods of sustained cognitive processing [19,48,49,50,51].

#### 2.4.10. Peg and Ball Task

In this computerized version of the executive function task subjects were presented with two configurations of three coloured balls (blue, green, red) on three pegs that each hold three balls. The subjects have to rearrange the balls, moving one ball at a time, from the starting configuration so that they match the position of the balls in the goal configuration. Subjects randomly completed 5 trials each which can be solved in 3, 4 and 5 moves respectively. Each trial generates scores for planning times prior to moving, time to complete and errors.

#### 2.4.11. Delayed Word Recall

The participant was again given 60 s to write down as many of the words presented previously as possible. Total number of correct responses and errors were recorded. 

#### 2.4.12. Delayed Word Recognition

The original 15 words plus 15 distractor words were presented one at a time in a random order. For each word, the participant indicated whether or not it was included in the original list of words by pressing appropriate “yes” and “no” keys as quickly as possible. Stimuli remained on screen until an appropriate response had been made. Accuracy and mean reaction time for correct responses were recorded.

#### 2.4.13. Delayed Picture Recognition

The original 15 pictures plus 15 distractor pictures were presented one at a time in a randomised order. For each picture participants indicated whether or not it was recognised as being from the original series by pressing appropriate “yes” and “no” keys as quickly as possible. Stimuli remained on screen until an appropriate response had been made. Accuracy and mean reaction time for correct responses were recorded. 

#### 2.4.14. Names-to-Faces Recall

The twelve original photographs presented at the outset were again presented on the screen, one at a time. Underneath each picture there was a list of 4 different first names and 4 different last names. For each photograph participants were instructed to choose the first and last name that was originally presented with the photograph. The numbers of correct responses for first and last names were recorded and collapsed to give an overall score for this task.

#### 2.4.15. Mood Measures

Two subjective ratings of mood were taken. A computerised version of the 16 Bond-Lader visual analogue scales was delivered via the COMPASS system prior to the start of each cognitive assessment. The results were combined to form 3 mood factors: “alert”, “calm” and “content” [52]. Participants also completed the Depression, Anxiety and Stress Scales (DASS) on arrival on each day. This 42-item self-report questionnaire requires participants to rate how much each statement of negative emotional state applied to them during the past week. This measure is designed to assess both current state and change in state over time [53].

### 2.5. Procedure

Participants attended the laboratory on 4 separate occasions (Introductory/screening visit, Practice Day, Day 1 and Day 56). Testing took place in a suite of testing facilities within the Brain Performance and Nutrition Research Centre, Northumbria University, with participants visually isolated from each other.

The Introductory visit to the laboratory comprised: briefing on the requirements of the study; the obtaining of informed consent; collection of demographic data; health screening; and training on the cognitive and mood measures.

Following the introductory visit participants attended the laboratory in an overnight fasted state at 8.30 a.m. on three separate occasions (Practice Day, Day 1 and Day 56). The Practice Day was a full facsimile of the subsequent visits but was conducted solely to familiarise the participants with the study procedures/assessments, allow for an assessment of the participants ability to adequately perform the cognitive tasks and mitigate any practice effects. On the Practice Day participants received a commercially available breakfast bar and the subsequent data were not entered into the analysis.

The assessment procedure on each day was identical: on arrival participants completed the paper and pencil Depression, Anxiety and Stress Scales (DASS) followed by a computerised cognitive assessment. Following this they consumed their treatment for that day. Two further cognitive assessments commenced at 40 and 160 min following consumption of the day’s treatment. The first of these assessments was designed to coincide with the known bioavailability of several components and the latter was timed to coincide with the natural decline in cognitive function typically seen in the late morning. The active study days, Day 1 and Day 56, differed only in that participants consumed their randomly allocated intervention (“nutrient enriched” or “placebo” breakfast bar) immediately after the baseline assessment. At the end of Day 1 they also took away a box containing 4 weeks supply of their daily intervention and a diary sheet to record their consumption of the breakfast bars. After 4 weeks participants returned to collect a further 28 days’ supply of breakfast bars and confirm continued compliance with the study procedures and inclusion/exclusion criteria with the researcher. At the end of Day 56 diary sheets and uneaten bars were scrutinised to confirm compliance. The Timelines and assessments on Day 1/Day 56 are shown in Figure 2B.

### 2.6. Statistics

#### 2.6.1. Acute and Acute/Chronic Superimposed

Change from baseline data were calculated for both the 40- and 160-min post-dose assessment on both Day 1 and Day 56 with reference to the corresponding pre-dose score on each day.

These data for each outcome were entered into three-way (treatment (nutrient enriched/placebo) × assessment (40/160 min) × day (1/56)) ANOVAs for those tasks that were completed once per assessment and four-way (treatment × repetition (1–4) × assessment × day) ANOVAs for those tasks from the Cognitive Demand Battery which were repeated four times per assessment.

By using this model any purely acute effect of treatment would be seen as a significant main effect of treatment and any acute effect that had been increased or attenuated by chronic supplementation (defined here as “acute/chronic superimposed”) would be seen as a significant treatment × day interaction. Higher order interactions would require further interpretation as necessary. 

#### 2.6.2. Pure Chronic Effects

Unadjusted data from each outcome from the baseline assessment on Day 1 and Day 56 were entered into a two-way (treatment × day) ANOVA for those tasks that were completed once per assessment and a three-way (treatment × repetition × day) ANOVA for those tasks from the Cognitive Demand Battery which were repeated four times per assessment.

Using this approach any chronic effect of treatment (with no contribution attributable to the Day 56 dose of treatment) would be evinced as a treatment × day interaction.

All ANOVA results for individual tasks that evinced a significant treatment related interaction effect were further explored with Bonferroni adjusted *t* tests (calculated using MSError from the ANOVA) comparing data from the two treatments as required. The stated probability values reflect the Bonferroni adjustment. Given the exploratory nature of the study no further adjustment was made for the number of outcomes overall. 

## 3. Results

### 3.1. Acute and Acute/Chronic Superimposed Effects

#### 3.1.1. Individual Cognitive Tasks and Mood Measures

**Figure 3:**
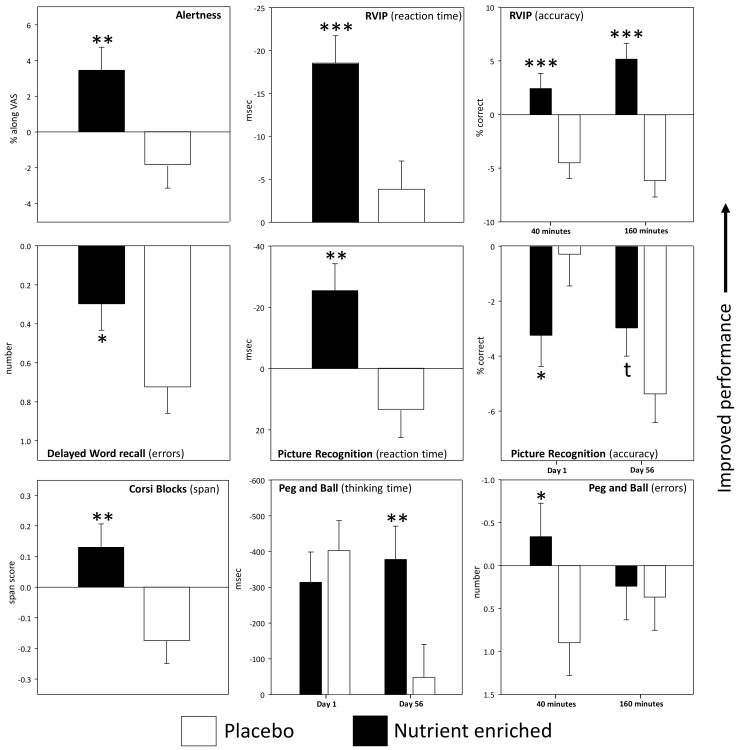
Effects of the nutrient enriched bars on performance of the tasks and mood scales that were repeated once per assessment. Data are baseline adjusted (to the day’s pre-treatment score) means (±SEM). Asterisks represent a significant difference between treatments in terms of main effects of treatment on the ANOVA or, for those measures that evinced an interaction, from Bonferroni adjusted post-hoc comparisons. * *p* < 0.05; ** *p* < 0.01; *** *p* < 0.001.

In terms of mood, there was a significant main effect of treatment on Bond-Lader ratings of “alertness” (*F*(1, 93) = 8.919, *p* = 0.004) with consumption of the nutrient enriched treatment associated with increased alertness across post-dose assessments on Day 1 and Day 56. Means (±SEM) are depicted graphically in Figure 3, with data from each assessment presented in Table 2.

Consumption of the nutrient enriched treatment was also associated with acute improvements (evinced as a main effect of treatment across Day 1 and Day 56) to tasks across cognitive domains. In terms of attention, there was a significant main effect of treatment with regards the accuracy of performing the Rapid Visual Information Processing (RVIP) task (*F*(1, 87) = 24.74, *p* < 0.001) with the nutrient enriched treatment associated with improved accuracy across post-dose assessments on Day 1 and Day 56. There was also a significant assessment x treatment interaction (*F*(1, 87) = 6.29, *p* = 0.014) with regards the same measure (Figure 3). Reference to the post-hoc comparisons showed that accuracy was improved during both assessments (40 min (t (87) = 4.65, *p* < 0.001), 160 min (t (93) = 7.59, *p* < 0.001)) with the interaction representing a numerically greater magnitude of improvement during the 160 min assessment. With regards the speed of performing the RVIP task, there was also a significant main effect of treatment (*F*(1, 87) = 10.29, *p* = 0.002) with the nutrient enriched treatment associated with increased speed across post-dose assessments on Day 1 and Day 56.

With regard to working memory, there was a significant main effect on the Corsi blocks span score (*F*(1, 93) = 8.49, *p* = 0.004), with the nutrient enriched treatment being associated with improved working memory capacity across post-dose assessments on Day 1 and Day 56. There was also a significant assessment × treatment interaction (*F*(1, 93) = 4.345, *p* = 0.04) with regards the accuracy of performing the Numeric Working Memory task. However, reference to comparisons between means showed that there were no significant differences between treatments at either assessment.

Episodic memory task performance was also improved by the nutrient enriched treatment, with main effects seen in terms of a reduced number of errors committed on the Delayed Word Recall task (*F*(1, 92) = 4.94, *p* = 0.029) and improved speed of performing the Picture Recognition task (*F*(1, 90) = 9.9, *p* = 0.002) across post-dose assessments on Day 1 and Day 56. However, with regards the latter task, there was also a significant treatment × day interaction (*F*(1, 90) = 6.15, *p* = 0.015) in terms of accuracy of performance. Reference to the means showed that this effect represented a decrement in performance on Day 1 (t (93) = 2.44, *p* < 0.05), with a trend towards improved performance on Day 56 (t (93) = 1.99, *p* < 0.1).

In terms of executive function, there was a significant treatment × day interaction (*F*(1, 93) = 6.33, *p* = 0.014) on the Peg and Ball task in terms of “thinking time”. Reference to the post-hoc comparisons showed that participants required a shorter amount of time to plan their moves following consumption of the nutrient enriched intervention but only on Day 56 (t (93) = 3.57, *p* < 0.01). However, there was also significant treatment × assessment interaction (*F*(1, 93) = 4.55, *p* = 0.036) with regards the number of errors committed during the same task. Reference to the post-hoc comparisons suggested that this effect represented an increased accuracy of performance following the nutrient enriched bar which was restricted to the 40 min post-dose assessment (t (93) = 2.55, *p* < 0.05).

Means (±SEM) for the measures from the tasks that were repeated once per assessment and which evinced a significant treatment related effect are depicted graphically in Figure 3. Means (±SEM) for each assessment on Day 1 and Day 56 are presented in Table 2.

#### 3.1.2. Cognitive Demand Battery (CDB)

Consumption of the nutrient enriched treatment was associated with acute improvements on all three tasks within the Cognitive Demand Battery. There was a main effect of treatment; representing increased correct subtractions following the nutrient enriched bars across post-dose assessments on Day 1 and Day 56, for both the Serial 3s and Serial 7s tasks (Serial 3s (*F*(1, 93) = 24.4, *p* < 0.001); Serial 7s (*F*(1, 93) = 13.4, *p* < 0.001)). There was also a main effect of treatment on both the accuracy of performing the Rapid Visual Information Processing task; with the nutrient enriched intervention leading to improvements in accuracy across Day 1 and Day 56 (*F*(1, 75) = 47.7, *p* < 0.001) and improved speed of performance (main treatment effect (*F*(1, 75) = 8.1, *p* = 0.006)). As well as these objective improvements in performance subjective ratings of mental fatigue (measured after each repetition of the three CDB tasks) was also significantly reduced across Days 1 and 56 following the nutrient enriched intervention (*F*(3, 279) = 22.5, *p* < 0.001).

**Figure 4:**
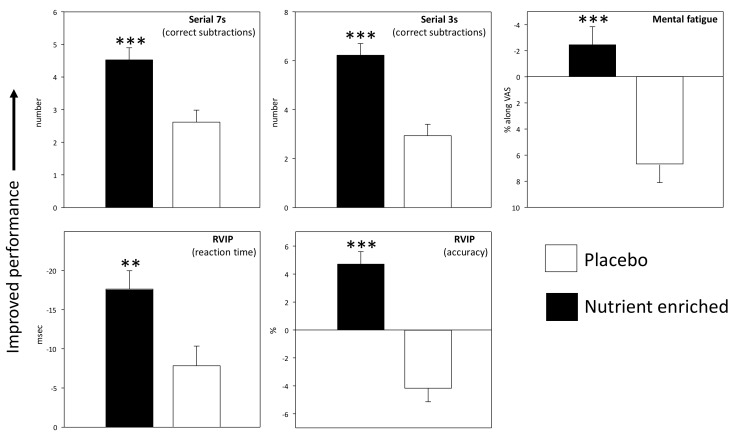
Effects of the nutrient enriched bars on performance of the Cognitive Demand Battery tasks that were repeated four times per assessment. Data are baseline adjusted (to the day’s pre-treatment score) means (±SEM). All of the effects were main effects of treatment across assessments and days. Asterisks represent a significant difference between treatments in terms of main effects of treatment on the ANOVA. * *p* < 0.05; ** *p* < 0.01; *** *p* < 0.001.

Means (±SEM) for the CDB tasks that evinced a significant treatment related effect are depicted graphically in Figure 4. Data (means ± SEM) from each repetition of the tasks during each assessment on Day 1 and Day 56 are presented in Table 3. 

### 3.2. Pure Chronic Effects

There were no significant differences in performance for any task during the pre-dose assessment on Day 56 that would indicate a chronic effect of treatment in the absence of any acute effects.

## 4. Discussion

The consumption of a nutrient enriched breakfast bar was associated with broad, significant, acute improvements in cognitive function across all cognitive domains, in comparison to placebo, on both Day 1 and Day 56 of an 8 weeks treatment regimen. Specifically, significant benefits were seen in terms of attention (RVIP), episodic memory (Delayed word recall, Picture recognition (speed)), working memory (Numeric working memory, Corsi Blocks, Serial subtractions) and executive function (Peg and Ball, Serial subtractions). In addition, subjective alertness and mental fatigue were improved. The only decrements in performance were seen in terms of a decrease in accuracy on the Picture recognition task on Day 1, with a trend towards the opposite effect on Day 56.

There was, however, no evidence of a simple chronic effect of 56 days of treatment with the nutrient enriched bars when the pre-treatment, baseline, performance data on Day 1 and Day 56 were compared. However, it should be noted that the statistical power of this comparison was somewhat lower than that of the acute/superimposed analysis and did not benefit from a baseline comparator. Having said this, the only evidence of differential acute effects between Day 1 and Day 56 which might represent a chronic/superimposed effect of the intervention was a significant interaction between day and treatment on the Peg and Ball task thinking time; showing that this measure was enhanced only on Day 56. Of course, one striking aspect of the current results is that, whilst there was no evidence of chronic effects, the acute effects of the nutrient enriched intervention was still apparent after 56 days of daily consumption, with no evidence of habituation to its effects.

Clearly, the demonstration of such pronounced benefits to cognitive function/mood raises the question of which component(s) are responsible. It is always tempting to single out caffeine, where it is present, as the major psychoactive component of any multi-ingredient intervention. However, this seems unlikely here for several reasons. The first is that the daily nutrient enriched intervention contained a low dose of 21.5 mg of caffeine, which equates to less than the caffeine in half a cup of tea and approximately a fifth of that present in a cup of filter coffee. Whilst a dose as low as 12.5 mg caffeine has been shown to exert some limited cognitive effects in a single study [54], the dose administered here was well below the typical psychoactive doses (75–200^+^ mg) employed in caffeine research. Whilst there has been little research assessing dose-responses to caffeine, it has previously been shown [55] that a 50 mg dose of caffeine increased subjective alertness but did not engender the beneficial effects on cognitive function and other aspects of mood, associated with two higher doses (150 mg and 450 mg) of caffeine. The second is that the effects seen here are also far more comprehensive than caffeine’s typical effects, which are generally restricted to increased alertness/arousal and improvements restricted to relatively simple attention/vigilance tasks. Caffeine does not usually have any effect on long-term (episodic) memory tasks and has inconsistent effects on working memory tasks; with evidence suggesting that it impairs the performance of more complex tasks [24,56,57]. With regards the working memory tasks utilized here, two previous experiments using the same Cognitive Demand Battery demonstrated that 33 mg, 38 mg and 46 mg of caffeine could improve performance on the RVIP but only in terms of accuracy of performance, with no improvements seen in terms of speed of performance of the RVIP or on the Serial 3s and Serial 7s tasks [51]. The serial subtraction tasks could best be described as complex, working memory/executive function tasks and caffeine would therefore not be expected to have an impact. Importantly, performance on both of these serial subtraction tasks was improved in the current study following consumption of the nutrient enriched bars, alongside improvements in both speed and accuracy of the RVIP task.

Naturally, this raises the question of which non-caffeine components might have been responsible for the striking findings here? The general assumption that vitamins and minerals have to be administered over weeks or months to have any physiological effect is not based on scientific evidence. Few studies have assessed the acute effects of micronutrients but, from those studies that have, there is good evidence to suggest that vitamins/minerals have physiological and brain function effects following a single dose [4]. For instance, single doses of vitamins B_9_, C, E and C/E combined have all been shown to increase vasodilation, as measured by peripheral flow mediated dilation, in groups with disease-related or experimentally induced endothelial dysfunction [30,31,32,33,34,35]. Similarly, single doses of calcium [36], calcium combined with vitamin D [37] and a multi-vitamin/mineral [38] have been shown to increase fat oxidation and, for the multivitamin/mineral, overall energy expenditure [38] as measured from exhaled pulmonary gas using Indirect Calorimetry. 

Naturally, any intervention that has an impact on metabolism or blood-flow will also inevitably have an impact on the brain; this being the most metabolically active organ in the body. More direct evidence of acute brain function effects of micronutrients include the demonstration of improvements in attention and semantic memory task performance following the first dose of a multivitamin/mineral administered to children [39] and modulation of regional brain activity during a task measuring focussed attention, as measured with fMRI [40] and cerebro-electrical activity (EEG-steady state visually evoked potentials) during an attention task [41] following a multi-vitamin/mineral. A recent study [38] also demonstrated increased cerebral blood-flow in the frontal cortex during task performance following a single dose of a multivitamin/mineral.

Beyond vitamins and minerals, the nutrient enriched bars also contained several other components that could plausibly have had an acute impact on performance. For instance, oral administration of high (~7–21 g) single doses of the amino-acid l-tyrosine, the direct precursor to the catecholamine neurotransmitters and trace amines, have been consistently shown to attenuate the cognitive and mood decrements associated with a range of stressors (e.g., sleep deprivation, cold, noise, physical and psychological stress) in humans [57,58,59,60,61,62,63,64,65]. More recent research has also shown that lower, single doses of 2 g of l-tyrosine can improve cognitive function in the absence of stressors [26,27,28,29], with some evidence that these effects are stronger for more cognitively demanding tasks [29,66]. Whilst a lower dose (279 mg) of l-tyrosine was administered here, there is, to date, no evidence that lower doses such as this would not be equally effective, and no data relating to the interactive effects of l-tyrosine and the other essential amino acids administered here (l-tryptophan (60 mg) and lysine (860 mg)). It may be relevant that the nutrient enriched bars also contained other components that have been shown to have acute beneficial effects on cognitive function, including whey protein [67,68] and cocoa-flavonols [19], although the quantities administered here were somewhat less than those investigated previously.

One further explanation of the striking improvements in cognitive function, alertness and mental fatigue seen here could be that the low dose of caffeine has evinced an interactive or synergistic effect with one or more of the many other potentially bioactive ingredients in the nutrient enriched product. A comparatively small but growing body of research suggests that caffeine’s effects are typically modulated by the presence of other bioactive components and vice versa. The most obvious example here is the differential effects of coffee and tea on psychological functioning which are mediated by both the varying caffeine levels and the presence, or not, of l-theanine [69]. This amino acid was present at 125 mg in the nutrient enriched bar, equivalent to the amount found in 5 cups of tea [70]. This tea-specific amino-acid has been shown to potentiate the effects of caffeine on cognitive function and mood [25] and abolish caffeine’s negative effects in terms of increased blood pressure [71] and decreased cerebral blood-flow [70]. Similarly, the co-administration of the amino acids taurine [72,73] and l-ornithine [74], the non-caffeine phytochemicals present in guarana [75], vitamins [40,41], glucose [76,77] and ephedrine [78] have also been shown to interact with caffeine with regards brain function. One final potential factor that might have had an impact on the results here may be the lengthy cognitive assessment and the resultant inculcation of mental fatigue. This may well have contributed to the pattern of results—indeed, the Cognitive Demand Battery is specifically designed to fatigue participants in order to provide a sensitive milieu for any treatment related effects. However, the pattern of cognitive improvements seen here included improvements to the performance of tasks from across the assessment, rather than just towards the later stages, suggesting that any improvements were not overly dependent on the participants’ level of mental fatigue.

Whilst the results here did not demonstrate any specific chronic effects of 8 weeks supplementation, this does not preclude the possibility that extended consumption of a nutrient enriched food such as this will have long term consequences for brain function. As an example, the nutrient enriched bars contained 1.4 g of omega-3 PUFAs, in the form of α-linolenic acid derived from the flax meal and walnut components. α-Linolenic acid can provide an important source of other bioactive omega-3 fatty acids such as EPA and DHA in the large proportion of the population that have no intake, or low intake, of these PUFAs. The mechanisms of action of omega-3 PUFAs are typically thought to relate to DHA’s role as a major component of the membrane phospholipids in the brain as well as its multifarious contributions to neural membrane structural properties and functions. Alternatively, the role of eicosanoid and docosanoid omega-3 PUFA derivatives as cellular mediators of inflammation, immune function, oxidative damage and vascular responses [10] are also thought to be key. Both of these mechanisms would become more relevant as the supplementation period continued. In line with this, whilst 3 months administration of omega-3 PUFAs has been shown to have no effect on cognitive function in healthy adults [79,80], 6 months administration did result in improved memory and attention task performance [23]. Interestingly, 3 months administration of DHA did increase haemodynamic responses in the prefrontal cortex during tasks but failed to result in performance or mood benefits [81,82]. A similar argument can also be made for the potential chronic effects of micronutrients; specifically, if their mechanisms of action involve the mitigation of, for instance, ongoing oxidative stress or homocysteine related neuronal damage, then their effects will only become apparent following a relatively long supplementation period.

## 5. Conclusions

In conclusion, a nutrient enriched breakfast bar, which contained potentially bioactive levels of vitamins/minerals, omega-3 PUFAs, amino acids, including l-theanine and caffeine, resulted in broad acute improvements in cognitive function and alertness that were sustained even after 56 days administration.

## Figures and Tables

**Table 1:** Demographics of the participants in the two treatment groups.

	Placebo Bar	Nutrient Enriched Bar
Number	48	47
Female/male	31/17	27/20
Age	24.5	25.1
Education (year)	17.2	17.5
Vegetables (portion/day)	3.5	3.3
Caffeine consumption (mg/day)	133	150
Blood pressure (sys/dia)	122/76	124/77
Heart rate (bpm)	69	69
Weight (Kg)	67.5	70.3
Height (cm)	170.5	170.6
Body mass index	23.2	24.1

There were no significant differences, on any factor, between the two groups (*p*’s all > 0.05).

**Table 2:** Data from the cognitive tasks with a single repetition during each assessment.

		*N*	Day 1						Day 56						ANOVA Sig. Effects
			Pre-Dose Baseline		40 min Post (Change)		160 min Post (Change)		Pre-Dose Baseline		40 min Post (Change)		160 min Post (Change)		
Alert % along VAS	Placebo	48	55.50	*2.15*	−1.61	*1.29*	−4.64	*1.69*	54.59	*2.29*	0.79	*1.58*	−2.02	*2.00*	Tr *p* = 0.004
	Nutrient+	47	54.20	*2.17*	4.49	*1.31*	4.62	*1.71*	54.72	*2.32*	3.42	*1.60*	1.36	*2.02*	
Content % along VAS	Placebo	48	59.85	*1.92*	0.49	*0.98*	−0.78	*1.39*	61.05	*1.96*	−0.31	*1.03*	−0.88	*1.49*	
	Nutrient+	47	61.07	*1.94*	1.93	*0.99*	2.59	*1.41*	60.41	*1.98*	1.03	*1.04*	0.72	*1.50*	
Calm % along VAS	Placebo	48	61.21	*1.70*	−0.83	*1.29*	−2.12	*1.15*	61.91	*1.96*	−3.08	*1.35*	−2.15	*1.51*	
	Nutrient+	47	62.33	*1.72*	−2.23	*1.30*	0.11	*1.16*	63.13	*1.98*	−4.60	*1.37*	−4.34	*1.52*	
Immediate word recall number correct	Placebo	48	7.23	*0.29*	−0.25	*0.29*	−0.62	*0.30*	7.71	*0.33*	−0.55	*0.32*	−0.65	*0.26*	
	Nutrient+	47	7.51	*0.30*	−0.62	*0.29*	−0.55	*0.30*	7.84	*0.33*	−0.31	*0.32*	−0.85	*0.27*	
Immediate word recall errors	Placebo	48	0.35	*0.09*	0.08	*0.12*	0.29	*0.13*	0.40	*0.11*	0.06	*0.14*	0.25	*0.15*	
	Nutrient+	47	0.40	*0.09*	0.19	*0.12*	0.11	*0.14*	0.49	*0.11*	−0.11	*0.14*	0.09	*0.15*	
Numeric working memory % correct	Placebo	48	95.72	*0.54*	−0.37	*0.53*	−0.33	*0.55*	95.51	*0.61*	−1.07	*0.64*	−0.32	*0.63*	Tr × Ass *p* = 0.04
	Nutrient+	47	95.68	*0.55*	−0.38	*0.54*	−0.40	*0.56*	95.93	*0.62*	0.12	*0.65*	−1.28	*0.64*	
Numeric working memory reaction time (msec)	Placebo	48	724.60	*21.16*	−33.93	*11.23*	−40.55	*12.09*	703.96	*19.24*	−16.01	*10.60*	−44.00	*11.81*	
	Nutrient+	47	710.97	*21.38*	−25.55	*11.35*	−40.75	*12.21*	711.91	*19.44*	−24.64	*10.71*	−41.06	*11.93*	
Choice reaction time % correct	Placebo	48	95.67	*0.46*	0.00	*0.46*	0.33	*0.48*	95.83	*0.44*	−0.17	*0.46*	0.66	*0.50*	
	Nutrient+	46	96.74	*0.47*	−0.44	*0.47*	−0.26	*0.49*	97.13	*0.45*	−0.31	*0.47*	−1.53	*0.51*	
Choice reaction time reaction time (msec)	Placebo	48	387.44	*6.81*	2.42	*5.22*	−2.23	*5.56*	386.11	*6.67*	10.23	*5.26*	13.93	*5.48*	
	Nutrient+	46	395.42	*6.96*	4.47	*5.34*	−4.07	*5.68*	398.75	*6.81*	3.69	*5.37*	−5.21	*5.60*	
Rapid Visual Inf Processing % correct	Placebo	44	61.31	*2.91*	−2.91	*1.93*	−3.95	*1.94*	59.26	*3.23*	−6.16	*1.78*	−8.43	*2.17*	Tr *p* < 0.001 Tr × Ass *p* = 0.014
	Nutrient+	46	58.91	*2.84*	2.94	*1.87*	6.47	*1.88*	56.36	*3.16*	1.96	*1.72*	3.91	*2.10*	
Rapid Visual Inf Processing reaction time (msec)	Placebo	44	470.3	*6.51*	3.16	*5.57*	−3.14	*5.56*	463.8	*10.57*	−10.10	*5.17*	−5.28	*5.45*	Tr *p* = 0.002
	Nutrient+	46	477.8	*6.36*	−21.68	*5.38*	−19.04	*5.37*	474.3	*10.33*	−15.98	*5.00*	−17.45	*5.27*	
Corsi Span span	Placebo	48	6.35	*0.13*	−0.42	*0.12*	−0.16	*0.13*	6.22	*0.14*	−0.06	*0.12*	−0.06	*0.13*	Tr *p* = 0.004
	Nutrient+	47	6.25	*0.13*	0.16	*0.12*	0.06	*0.13*	6.05	*0.14*	0.16	*0.12*	0.16	*0.13*	
Peg and Ball thinking time (msec)	Placebo	48	3049	*176.3*	−415.5	*86.2*	−389.6	*102.2*	2682	*170.5*	64.8	*100.2*	−160.9	*105.6*	Tr × Day *p* = 0.014
	Nutrient+	47	2586	*178.2*	−255.3	*87.1*	−372.5	*103.2*	2661	*172.3*	−345.2	*101.3*	−410.6	*106.7*	
Peg and Ball completion time (msec)	Placebo	48	7597	*260.8*	−775.7	*150.0*	−990.2	*173.2*	7145	*195.5*	−100.9	*117.0*	−469.5	*141.1*	
	Nutrient+	47	7438	*263.6*	−818.7	*151.6*	−1034.2	*175.1*	7070	*197.6*	−498.0	*118.2*	−742.3	*142.6*	
Peg and Ball number of errors	Placebo	48	2.40	*0.44*	0.48	*0.55*	0.08	*0.56*	2.81	*0.37*	1.33	*0.50*	0.67	*0.51*	Tr × Ass *p* = 0.036
	Nutrient+	47	3.62	*0.44*	−1.32	*0.56*	−0.45	*0.57*	2.23	*0.37*	0.64	*0.50*	0.94	*0.51*	
Delayed word recall number correct	Placebo	47	4.19	*0.31*	−1.46	*0.33*	−2.28	*0.34*	5.25	*0.34*	−2.21	*0.33*	−2.36	*0.33*	
	Nutrient+	47	4.69	*0.31*	−2.38	*0.33*	−2.14	*0.34*	5.05	*0.34*	−1.83	*0.33*	−2.00	*0.33*	
Delayed word recall errors	Placebo	47	0.96	*0.15*	0.70	*0.21*	0.94	*0.23*	0.75	*0.14*	0.68	*0.21*	0.57	*0.20*	Tr *p* = 0.029
	Nutrient+	47	0.83	*0.15*	0.55	*0.21*	0.47	*0.23*	0.92	*0.14*	−0.02	*0.21*	0.19	*0.20*	
Name to Face % correct	Placebo	48	37.50	*3.06*	−7.20	*2.86*	−9.47	*3.24*	47.57	*3.37*	−7.96	*3.70*	−11.55	*3.12*	
	Nutrient+	45	36.11	*3.16*	−4.06	*3.04*	−6.84	*3.44*	42.96	*3.48*	−4.91	*3.93*	−9.40	*3.32*	
Name to Face reaction time (msec)	Placebo	48	5427	*320.3*	−377.7	*327.0*	−376.0	*375.8*	5442	*221.3*	−177.4	*207.9*	−461.1	*214.5*	
	Nutrient+	45	5514	*330.8*	−422.7	*347.3*	−805.7	*399.1*	5035	*228.5*	−300.8	*220.9*	−493.7	*227.9*	
Picture recognition % correct	Placebo	45	90.59	*1.29*	−0.37	*1.28*	−0.22	*1.29*	94.82	*1.05*	−5.41	*1.23*	−5.33	*1.24*	Tr × Ass *p* = 0.015
	Nutrient+	47	89.43	*1.26*	−2.65	*1.25*	−3.83	*1.26*	91.12	*1.02*	−2.96	*1.21*	−2.98	*1.21*	
Picture recognition reaction time (msec)	Placebo	45	767.9	*16.09*	20.48	*15.01*	18.75	*13.54*	788.8	*17.39*	17.79	*14.57*	−2.63	*14.02*	Tr *p* = 0.002
	Nutrient+	47	798.4	*15.75*	−15.03	*14.68*	−30.96	*13.24*	809.6	*17.02*	−29.32	*14.26*	−26.74	*13.72*	
Word recognition % correct	Placebo	46	74.71	*1.42*	−1.20	*1.41*	−4.34	*1.46*	79.06	*1.51*	−1.18	*1.40*	−2.08	*1.51*	
	Nutrient+	44	77.17	*1.46*	−3.76	*1.44*	−2.93	*1.49*	80.23	*1.55*	−0.99	*1.43*	−2.27	*1.55*	
Word recognition reaction time (msec)	Placebo	46	801.2	*18.23*	−17.48	*14.55*	−29.54	*17.10*	786.1	*20.86*	7.54	*18.72*	−58.10	*21.75*	
	Nutrient+	44	800.6	*18.64*	3.45	*14.88*	−32.04	*17.48*	780.7	*21.33*	14.21	*19.14*	14.35	*22.24*	

Data are means (+SEM, in italics) and comprise raw data from the pre-dose baseline assessment and “change from baseline” (adjusted to the days’ corresponding pre-dose score) data for the 40 min and 160 min post-dose assessments. Tr = main effect of treatment; Tr × Ass = treatment × assessment interaction; Tr × Day = treatment × day interaction—all from the ANOVA.

**Table 3:** Data from the Cognitive Demand Battery tasks that were repeated four times during each assessment.

			Pre-Dose Baseline								40 min Post-Dose (Change)								60 min Post-Dose (Change)								ANOVA Sig. Effects
	Day		Rep 1		Rep 2		Rep 3		Rep 4		Rep 1		Rep 2		Rep 3		Rep 4		Rep 1		Rep 2		Rep 3		Rep 4		
RVIP % correct	Day 1	Plac	63.33	*3.34*	60.1	*3.34*	57.92	*3.41*	55.5	*3.65*	−4.31	*1.92*	−3.82	*1.81*	−3.33	*1.77*	−2.50	*2.38*	−4.44	*1.95*	−5.76	*1.96*	−5.63	*2.12*	0.35	*2.08*	Treatment *p* < 0.001
		Nut+	60.18	*3.13*	58.7	*3.13*	56.10	*3.20*	52.8	*3.42*	3.35	*1.80*	5.30	*1.70*	6.28	*1.66*	7.44	*2.23*	4.27	*1.82*	−0.55	*1.84*	0.73	*1.98*	3.90	*1.95*	
	Day 56	Plac	59.17	*3.28*	54.4	*3.38*	54.31	*3.63*	51.1	*3.36*	−7.08	*2.11*	−2.22	*1.91*	−7.36	*2.04*	−2.01	*2.24*	−6.67	*1.92*	−4.83	*2.13*	−3.82	*1.99*	−3.54	*2.44*	
		Nut+	55.49	*3.08*	51.6	*3.17*	50.37	*3.41*	49.1	*3.15*	6.77	*1.98*	4.15	*1.79*	3.96	*1.92*	8.05	*2.09*	3.41	*1.80*	6.83	*1.99*	5.49	*1.87*	6.16	*2.28*	
RVIP Reaction time msec	Day 1	Plac	489.54	*8.75*	489	*8.32*	490	*8.89*	495	*7.99*	−3.07	*6.22*	−8.94	*5.83*	−11.7	*6.74*	−7.21	*6.20*	−9.49	*6.44*	−17.8	*5.64*	−12.8	*6.46*	−9.38	*7.15*	Treatment *p* < 0.006
		Nut+	486.76	*8.20*	483	*7.80*	483	*8.33*	483	*7.49*	−16.2	*5.83*	−18.3	*5.46*	−12.7	*6.32*	−7.7	*5.81*	−20.8	*6.04*	−17.8	*5.29*	−13.5	*6.06*	−8.32	*6.70*	
	Day 56	Plac	486.55	*7.84*	486	*8.48*	491	*8.37*	495	*7.65*	0.05	*5.36*	−1.91	*5.48*	3.20	*6.35*	−8.26	*6.65*	−10.3	*5.94*	−6.47	*5.22*	−3.93	*6.70*	−17.4	*7.14*	
		Nut+	488.00	*7.34*	497	*7.95*	502	*7.84*	493	*7.17*	−13.3	*5.02*	−23.5	*5.14*	−28.3	*5.95*	−14.6	*6.23*	−19.5	*5.57*	−29.6	*4.89*	−19.6	*6.27*	−17.9	*6.69*	
RVIP False alarms	Day 1	Plac	0.81	*0.16*	0.81	*0.15*	0.92	*0.18*	0.78	*0.14*	−0.08	*0.16*	0.10	*0.20*	−0.08	*0.18*	−0.08	*0.16*	−0.06	*0.18*	0.00	*0.19*	−0.28	*0.21*	−0.19	*0.18*	Treatment *p* < 0.005
		Nut+	0.42	*0.15*	0.46	*0.14*	0.34	*0.17*	0.34	*0.13*	0.24	*0.15*	0.15	*0.19*	0.02	*0.17*	0.27	*0.15*	0.17	*0.17*	0.37	*0.18*	0.37	*0.20*	0.49	*0.17*	
	Day 56	Plac	0.92	*0.18*	0.83	*0.14*	0.78	*0.15*	0.69	*0.14*	−0.03	*0.21*	−0.08	*0.19*	−0.08	*0.24*	−0.14	*0.20*	−0.28	*0.21*	−0.28	*0.15*	−0.14	*0.18*	0.22	*0.18*	
		Nut+	0.73	*0.17*	0.56	*0.13*	0.56	*0.14*	0.59	*0.13*	−0.12	*0.20*	0.07	*0.17*	0.27	*0.22*	0.15	*0.18*	−0.05	*0.20*	0.00	*0.14*	0.07	*0.16*	−0.05	*0.17*	
Serial 3s Number correct	Day 1	Plac	44.60	*2.17*	46.42	*2.20*	45.69	*2.03*	44.90	*2.18*	4.71	*1.00*	1.46	*1.04*	1.60	*1.07*	3.06	*1.09*	3.60	*1.10*	1.46	*1.04*	3.15	*1.30*	1.52	*1.25*	Treatment *p* < 0.001
		Nut+	46.68	*2.20*	49.30	*2.22*	47.77	*2.05*	48.70	*2.20*	7.60	*1.01*	3.94	*1.05*	5.28	*1.08*	5.02	*1.10*	7.09	*1.11*	4.45	*1.05*	4.36	*1.31*	4.34	*1.26*	
	Day 56	Plac	43.48	*2.40*	47.65	*2.28*	45.73	*2.30*	45.58	*2.36*	6.48	*1.52*	2.06	*1.27*	3.33	*1.11*	−0.79	*1.38*	7.90	*1.64*	1.81	*1.16*	3.15	*1.40*	2.46	*1.31*	
		Nut+	45.75	*2.42*	48.11	*2.31*	48.49	*2.32*	48.38	*2.39*	9.85	*1.54*	7.74	*1.28*	6.15	*1.12*	5.13	*1.39*	11.30	*1.66*	5.26	*1.17*	5.81	*1.41*	6.28	*1.32*	
Serial 3s Number of errors	Day 1	Plac	2.15	*0.30*	2.06	*0.37*	2.42	*0.33*	2.50	*0.38*	0.73	*0.44*	0.63	*0.46*	0.02	*0.42*	0.00	*0.40*	0.42	*0.37*	0.69	*0.38*	0.19	*0.39*	0.29	*0.49*	
		Nut+	2.77	*0.30*	2.64	*0.38*	2.72	*0.33*	2.72	*0.39*	0.00	*0.45*	0.15	*0.46*	0.11	*0.42*	0.28	*0.41*	−0.13	*0.37*	−0.26	*0.38*	−0.68	*0.40*	−0.11	*0.50*	
	Day 56	Plac	2.96	*0.37*	2.46	*0.35*	2.67	*0.36*	2.56	*0.35*	0.63	*0.47*	0.19	*0.37*	−0.17	*0.46*	0.58	*0.47*	−0.06	*0.47*	−0.17	*0.39*	−0.33	*0.39*	−0.19	*0.43*	
		Nut+	2.30	*0.37*	2.45	*0.35*	2.36	*0.36*	2.57	*0.35*	0.94	*0.47*	−0.30	*0.38*	0.55	*0.47*	0.40	*0.48*	0.49	*0.47*	0.38	*0.39*	−0.02	*0.40*	−0.15	*0.44*	
Serial 7s number correct	Day 1	Plac	25.94	*1.51*	26.42	*1.52*	26.81	*1.58*	26.40	*1.53*	2.83	*0.75*	2.38	*0.79*	1.56	*0.84*	1.58	*0.79*	2.50	*0.79*	2.90	*0.88*	3.17	*0.88*	3.92	*0.81*	Treatment *p* < 0.001
		Nut+	28.00	*1.53*	28.43	*1.53*	29.40	*1.60*	29.64	*1.55*	4.68	*0.76*	4.00	*0.80*	4.21	*0.85*	4.06	*0.80*	5.87	*0.80*	4.40	*0.89*	3.49	*0.89*	3.83	*0.82*	
	Day 56	Plac	26.58	*1.48*	26.17	*1.57*	26.98	*1.50*	27.15	*1.61*	2.25	*0.88*	2.52	*0.91*	1.21	*0.91*	1.13	*1.06*	4.33	*0.97*	4.10	*1.09*	2.92	*0.90*	2.54	*0.95*	
		Nut+	27.96	*1.50*	29.53	*1.59*	29.92	*1.52*	28.51	*1.62*	5.21	*0.89*	3.62	*0.92*	3.11	*0.91*	4.68	*1.07*	7.23	*0.98*	4.89	*1.10*	3.79	*0.91*	5.30	*0.96*	
Serial 7s number of errors	Day 1	Plac	2.25	*0.34*	2.33	*0.34*	2.71	*0.35*	2.31	*0.37*	0.94	*0.44*	0.10	*0.43*	0.38	*0.42*	0.69	*0.46*	0.75	*0.36*	0.60	*0.42*	−0.17	*0.43*	0.50	*0.42*	
		Nut+	2.26	*0.35*	2.81	*0.35*	2.89	*0.35*	3.21	*0.38*	0.70	*0.45*	0.68	*0.44*	0.23	*0.43*	−0.26	*0.46*	0.32	*0.36*	−0.32	*0.43*	0.38	*0.44*	0.17	*0.42*	
	Day 56	Plac	2.44	*0.32*	2.69	*0.34*	2.50	*0.30*	2.83	*0.40*	0.88	*0.35*	−0.10	*0.41*	0.83	*0.40*	0.15	*0.40*	0.31	*0.35*	−0.27	*0.39*	0.58	*0.35*	0.23	*0.41*	
		Nut+	2.32	*0.32*	2.62	*0.34*	2.92	*0.30*	3.75	*0.41*	0.79	*0.35*	0.04	*0.41*	0.09	*0.41*	−0.53	*0.41*	0.38	*0.36*	0.53	*0.39*	0.02	*0.35*	−0.87	*0.41*	
Fatigue % along VAS	Day 1	Plac	56.29	*2.48*	59.35	*2.80*	62.29	*2.84*	65.08	*2.93*	3.81	*2.42*	6.58	*2.27*	5.19	*2.36*	3.90	*2.16*	8.40	*2.43*	10.48	*2.22*	10.04	*2.14*	7.10	*1.90*	Treatment *p* < 0.001
		Nut+	57.89	*2.51*	62.83	*2.83*	67.38	*2.87*	70.45	*2.96*	−5.60	*2.44*	−7.09	*2.30*	−8.96	*2.39*	−11.5	*2.18*	−1.43	*2.45*	0.79	*2.24*	0.94	*2.16*	−0.28	*1.92*	
	Day 56	Plac	51.88	*2.51*	58.73	*2.46*	63.31	*2.55*	66.25	*2.72*	8.10	*2.40*	6.19	*2.19*	4.79	*2.05*	3.85	*1.97*	10.67	*2.47*	9.71	*2.31*	5.15	*2.39*	3.90	*2.27*	
		Nut+	57.87	*2.53*	65.45	*2.49*	70.47	*2.57*	74.87	*2.75*	−0.98	*2.42*	−3.40	*2.21*	−4.53	*2.07*	−6.28	*1.99*	5.17	*2.50*	3.49	*2.34*	1.53	*2.41*	−1.28	*2.30*	

Data are means (±SEM, in italics) and comprise raw data from the pre-dose baseline assessment and “change from baseline” (adjusted to the days’ corresponding pre-dose score) data for the 40 min and 160 min post-dose assessments. Sample sizes were 77 (placebo 36) for the Rapid Visual Information Processing task (RVIP) and 95 (placebo 48) for the Serial 3s, 7s and fatigue. Treatment = main effect of treatment from the ANOVA; Plac = placebo; Nut+ = nutrient enriched; RVIP = Rapid Visual Information Processing task; Rep = repetition of task.
